# Plasma levels of leptin, omentin, collagenous repeat-containing sequence of 26-kDa protein (CORS-26) and adiponectin before and after oral glucose uptake in slim adults

**DOI:** 10.1186/1475-2840-6-7

**Published:** 2007-02-20

**Authors:** Sylvia Wurm, Markus Neumeier, Johanna Weigert, Andreas Schäffler, Christa Buechler

**Affiliations:** 1 Department of Internal Medicine I, Regensburg University Hospital, D-93042 Regensburg, Germany

## Abstract

**Background:**

Adipose tissue secreted proteins are collectively named adipocytokines and include leptin, adiponectin, resistin, collagenous repeat-containing sequence of 26-kDa protein (CORS-26) and omentin. Several of these adipocytokines influence insulin sensitivity and glucose metabolism and therefore systemic levels may be affected by oral glucose uptake. Whereas contradictory results have been published for leptin and adiponectin, resistin has not been extensively investigated and no reports on omentin and CORS-26 do exist.

**Methods:**

Therefore the plasma levels of these proteins before and 120 min after an oral glucose load were analyzed in 20 highly-insulin sensitive, young adults by ELISA or immunoblot.

**Results:**

Circulating leptin was reduced 2 h after glucose uptake whereas adiponectin and resistin levels are not changed. Distribution of adiponectin and CORS-26 isoforms were similar before and after glucose ingestion. Omentin is highly abundant in plasma and immunoblot analysis revealed no alterations when plasma levels before and 2 h after glucose intake were compared.

**Conclusion:**

Taken together our data indicate that only leptin is reduced by glucose uptake in insulin-sensitive probands whereas adiponectin and resistin are not altered. CORS-26 was demonstrated for the first time to circulate as high molecular weight form in plasma and like omentin was not influenced by oral glucose load. Omentin was shown to enhance insulin-stimulated glucose uptake but systemic levels are not correlated to postprandial blood glucose.

## Background

Several adipose tissue derived proteins have a major impact on the regulation of energy homeostasis and therefore are in the focus of current research. Leptin, in the hypothalamus, regulates food uptake and in addition has numerous effects on peripheral cells and organs including the regulation of immune cells, pancreatic beta cells, adipocytes, and muscle and liver insulin sensitivity [1]. Adipocytokines may also exert a metabolic role in postprandial hyperglycemia and therefore, systemic leptin was investigated in several studies. Leptin concentrations during oral glucose tolerance test (OGTT) in normal weight women were found unchanged whereas in obese women an increase [2,3] or no alterations have been described [4]. In insulin-sensitive men, a reduction of leptin was detected in lean and obese probands whereas in the insulin-resistant state, leptin did not change [5].

While systemic leptin is increased in obesity, adiponectin is reduced [6]. Adiponectin is known to exert anti-inflammatory and insulin-sensitizing effects but in addition, proinflammatory activities have been described [6-8]. Adiponectin circulates in blood as trimers, hexamers, and higher molecular weight (HMW) complexes [6] and recent studies indicate that the HMW adiponectin is the active form of the protein [9]. Total circulating adiponectin was significantly increased in women with polycystic ovary syndrome at 120 min of OGTT [10] whereas it was not altered in normal glucose tolerant, impaired glucose tolerant and type 2 diabetic probands 2 h after glucose ingestion [11]. The adiponectin paralog CORS-26 (collagenous repeat-containing sequence of 26 kDa protein) is produced in adipocytes and monocytes and has antiinflammatory properties [12]. Although in vitro data suggest that CORS-26 is a secreted protein [12], CORS-26 in human plasma has not been investigated so far.

Resistin, initially identified in mice as an adipose tissue derived protein, has been associated with the low grade inflammation and insulin resistance in type 2 diabetic patients [13]. Although in rodents resistin cleary disturbs glucose and lipid homeostasis, its role in humans is unclear [14]. So far no association of circulating resistin and glucose homeostasis has been identified and healthy controls even had significantly higher systemic resistin levels than patients with type 1 and type 2 diabetes [15]. Systemic resistin was determined after oral glucose load and was not significantly altered during OGTT [10].

Omentin is highly abundant in the stroma vascular fraction of visceral fat and was detected in human serum by immunoblot [16]. Recombinant omentin enhances insulin-stimulated glucose uptake in adipocytes [16] but so far it was not analyzed whether systemic omentin is affected by acute hyperglycemia.

The adipose tissue secreted proteins described above were investigated in 20 young, insulin-sensitive probands after an overnight fast and 2 h following glucose uptake. The data obtained may help to further clarify the physiological regulation of well described plasma proteins like leptin and adiponectin but in addition reveal new insights on the abundance of resistin, omentin and CORS-26 in the plasma of young adults before and after glucose uptake.

## Methods

### Subjects

Twenty healthy subjects (6 females and fourteen males, weight range: 55–86 kg, mean BMI 22.5 ± 2 kg/m^2^, mean age 22.2 ± 0.5 years) participated in the study. A baseline blood sample was taken after overnight fasting. They were given 75 g glucose dissolved in 300 ml water and a second blood sample was taken 2 h later. The study protocol was approved by the local ethics committee and was carried out in accordance with the Helsinki guidelines. All probands gave written informed consent to participate in the study.

### Reagents

DuoSet ELISA Development System for human resistin, leptin and adiponectin as well as CORS-26 and adiponectin antibodies were purchased from R&D Systems (Wiesbaden-Nordenstadt, Germany). Insulin ELISA was from Mercodia (Uppsala, Sweden). Omentin antibody was raised in rabbits with the use of a peptide (IYQKYPVKYGEGKC) for immunization.

### SDS-PAGE and immunoblotting

The plasma was diluted 1:1,000 in PBS for the detection of adiponectin, 1:200 for CORS-26 and 1:50 for omentin immunoblots. Proteins were separated by SDS-polyacrylamide gel electrophoresis and were transferred to PVDF membranes. Incubations with antibodies were performed in 5% nonfat dry milk in PBS, 0.1% Tween. Detection of the immune complexes was carried out with the ECL Western blot detection system (Amersham Pharmacia, Deisenhofen, Germany).

### In-vitro translation of omentin

Full-length human omentin (Genbank acc. no. CB266342) was amplified with the primers omentin_uni: 5'-atgaaccaactcagcttcctgct-3' and omentin_rev: 5'-acgatagaatagaagcacagca-3' from human adipose tissue mRNA and was cloned into the expression vector pcDNA3.1/V5-His^(C) ^TOPO TA^(R) ^(Invitrogen, Karlsruhe, Germany) and sequenced. In-vitro translation was performed with the TNT^® ^T7 Quick Coupled Transcription/Translation System (Promega, Mannheim, Germany).

### ELISA

The ELISAs were performed as recommended by the distributor.

### Statistics

Data are presented as mean ± standard deviation (SPSS 12.0 for Windows). Statistical differences were analyzed by paired, two-tailed Student's t test and a value of p < 0.05 was regarded as statistically significant.

## Results

### Oral glucose tolerance test (OGTT)

Twenty healthy subjects participated in the study and blood glucose in the fasted state was 88 ± 7 mg/dl (reference value 70 to 110 mg/dl) and 2 h after the glucose challenge 79 ± 14 mg/dl (reference value: less than 140 mg/dl). Insulin was 2.4 ± 1 mU/l before and 7.4 ± 3.7 mU/l 2 h after oral glucose intake. The Homeostasis Model Assessment (HOMA) Index was 0.5 ± 0.2 (reference value < 1) indicating a high insulin sensitivity of the probands who participated in the current study.

### Determination of plasma leptin and adiponectin

**Figure 1:**
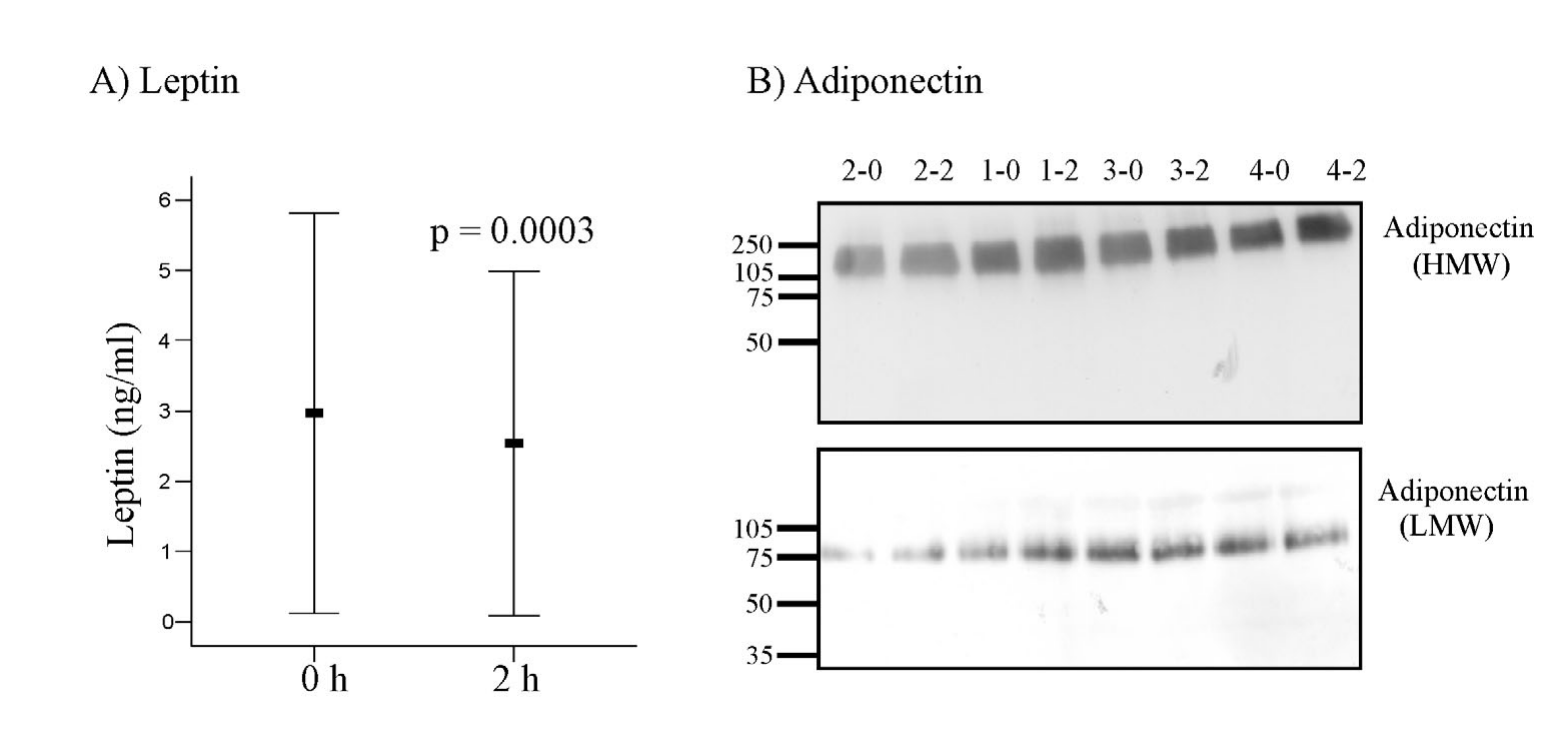
**Leptin and adiponectin in fasting plasma and plasma after glucose challenge**. (A) Leptin was determined in the plasma of 20 probands after an overnight fast and 2 h after glucose uptake by ELISA (B) Adiponectin was analyzed by non-denaturing SDS-PAGE (high-molecular weight, HMW) and denaturing SDS-PAGE (low-molecular weight, LMW) and a representative result after fasting (0) and 2 h (2) after glucose uptake is shown for probands 1 to 4. The molecular mass of standard proteins in kDa is indicated on the left side of the immunoblot.

Leptin was determined in the plasma of the 20 probands in the fasted state and was 3.0 ± 2.8 ng/ml and 2 h after glucose challenge it reached levels of 2.5 ± 2.5 ng/ml (p = 0.0003) (Figure 1A). Leptin in females in the fasted state was 6.7 ± 2.1 ng/ml, and 2 h after glucose challenge it was 5.7 ± 1.9 ng/ml (p = 0.0006). In males 1.3 ± 0.5 ng/ml were detected in the morning and 1.1 ± 0.6 ng/ml (p = 0.0005) postprandial. Leptin is higher abundant in women and this was confirmed in our study group (p < 0.0001).

Adiponectin was significantly higher in females with 21.3 ± 11.8 μg/ml when compared to males with 10.1 ± 3.4 μg/ml. In the fasted state, adiponectin was 13.5 ± 8.5 μg/ml and 13.1 ± 6.7 μg/ml 2 h after glucose uptake (p = 0.27) when the plasma of all donors was compared. Similar results were obtained when men and women were analyzed separately (not shown). Besides total circulating adiponectin, the levels of the HMW adiponectin isoforms were analyzed by immunoblot. Plasma samples were separated by SDS-PAGE under non-reducing conditions and HMW-APM was detected in all plasma samples (Figure 1B). Reducing conditions convert the HMW-APM to a protein subunit of about 75 kDa most likely resembling trimeric APM and similar amounts were detected in the plasma before and after glucose ingestion (Figure 1B).

### Determination of plasma resistin

Resistin was 1.77 ± 0.57 ng/ml before and 1.77 ± 0.59 ng/ml after glucose challenge (p = 0.47) and similar data were obtained when resistin plasma samples from men and women were calculated separately (not shown).

### Analysis of CORS-26 by immunoblot

**Figure 2:**
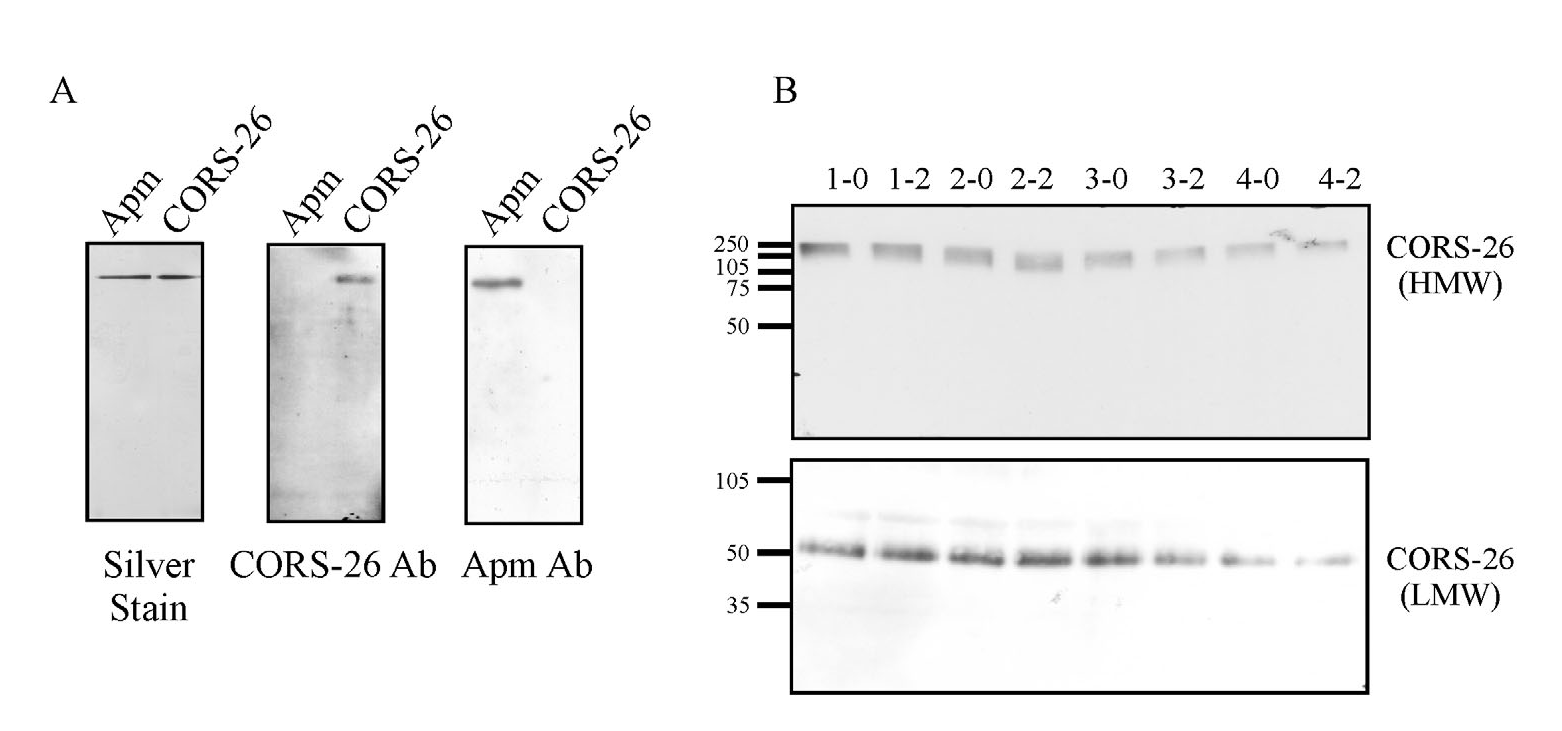
**Specificity of the CORS-26 antiserum and CORS-26 in fasting plasma and plasma after glucose challenge**. (A) Recombinant adiponectin (Apm) and CORS-26 on a silver-stained SDS-PAGE gel and upon detection by the respective antibodies using immunoblot. (B) High-molecular weight (HMW) CORS-26 was analyzed by non-denaturing SDS-PAGE and low-molecular weight (LMW) CORS-26 by denaturing SDS-PAGE and a representative result of 4 donors (1 to 4) after fasting (0) and 2 h (2) after glucose uptake is shown. The molecular mass of standard proteins in kDa is indicated on the left side of the immunoblot.

Specificity of the CORS-26 antibody was analyzed by immunoblot using recombinant adiponectin and recombinant CORS-26 produced in insect cells [7,12]. 100 ng of protein was separated by SDS-PAGE and silver staining of the gel revealed similar amounts of protein (Figure 2A). The membranes were hybridized with adiponectin or CORS-26 antibody and each antiserum revealed a specific signal with the respective protein (Figure 2A).

Plasma of the 20 probands was analyzed by immunoblot using the CORS-26 antibody and CORS-26 was found highly abundant. Quantification of the immunoblots using OptiQuant software revealed a 2-fold difference in CORS-26 plasma levels when all samples were compared (not shown).

CORS-26 was similarly abundant before and after glucose uptake but immunoblot is not suitable to detect changes of less than 20% in protein levels and therefore alteration below 20% can not be excluded (Figure 2B). Plasma samples were separated by SDS-PAGE under non-reducing conditions and CORS-26 was detected in all plasma samples as a high molecular weight complex of about 180 to 250 kDa (Figure 2B). Reducing conditions convert the HMW-CORS-26 to a protein subunit of about 60 kDa that may resemble monomeric or dimeric protein and similar amounts were detected in the plasma before and after glucose ingestion (Figure 2B).

### Analysis of omentin by immunoblot

**Figure 3:**
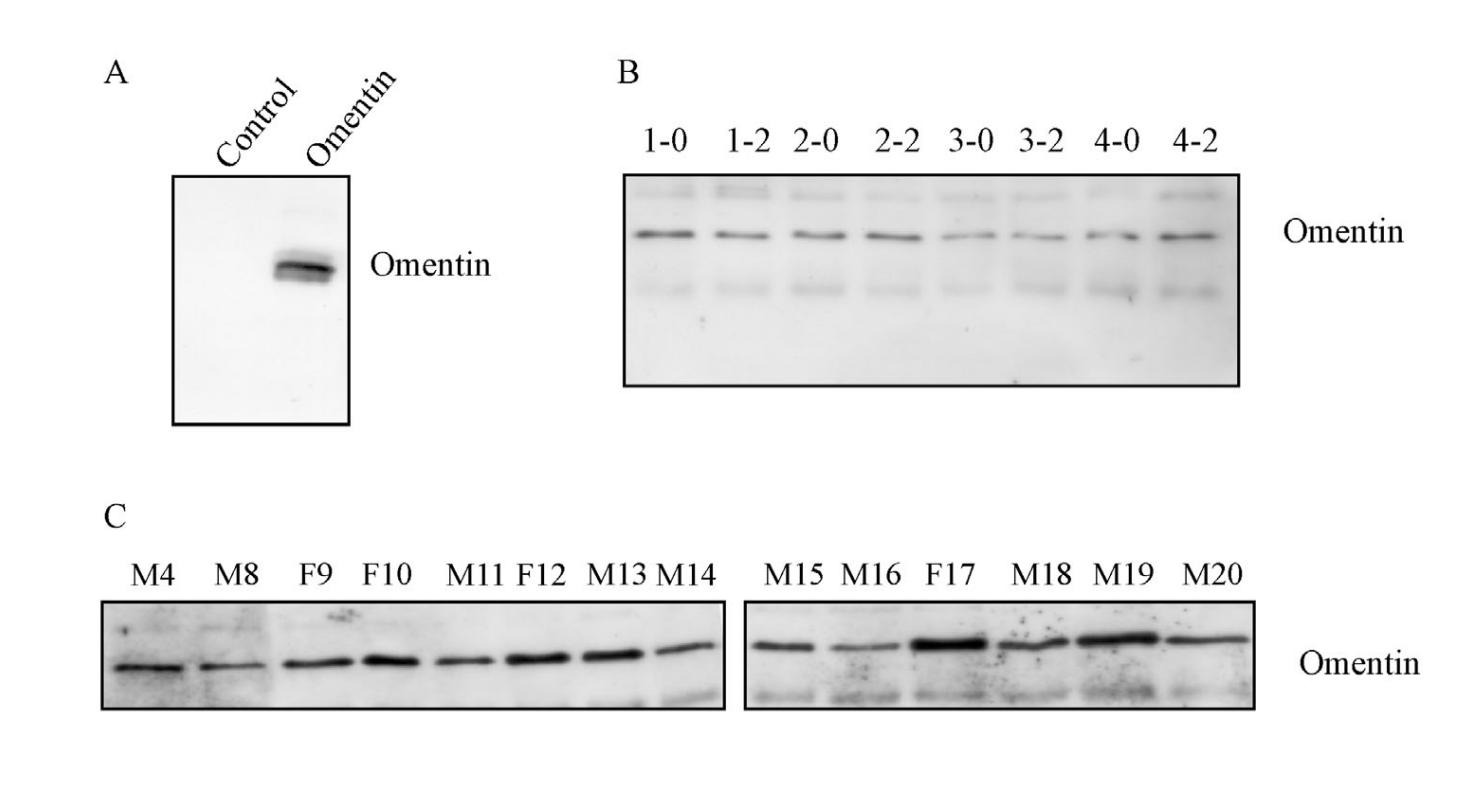
**Specificity of the omentin antiserum and omentin in fasting plasma and plasma after glucose challenge**. (A) In vitro translated omentin and a control protein were used to determine the specificity of the omentin antiserum. (B) Omentin was analyzed in the plasma of all probands and a representative result of the probands 1 to 4 after an overnight fast (0) and 2 h (2) after glucose uptake by immunoblot is shown. (C) Omentin in the plasma of women (F9, F10, F12, F17) and men (M4, M8, M11, M13, M14, M15, M18, M19, M20) was determined by immunoblot.

In vitro translated omentin and a control protein with a C-terminal V5 tag were used for immunoblot using an anti-V5 antibody (not shown) or anti-omentin antibody (Figure 3A). Anti-omentin antibody specifically detects omentin protein and therefore was used for immunoblots. Plasma before and after glucose uptake was analyzed and omentin with a molecular weight of 30 kDa was found similar abundant and a representative result is shown in figure 3B. Serial dilution of plasma revealed that a difference of 20 % can be detected by immunoblot and the subsequent quantification of the signals with OptiQuant software (not shown).

A high variability of omentin plasma levels was identified when plasma of different donors was compared and quantification of the immunoblot using OptiQuant software revealed an up to 5.8-fold difference. However, these variations were not related to the gender of the plasma donors (Figure 3C). Pearson correlation did not identify a correlation of plasma omentin levels with fasting or postprandial blood glucose or changes in blood glucose when fasting values and values 2 h after glucose ingestion were calculated (not shown).

## Discussion

Adipose tissue secretes a variety of molecules into the circulation. These adipocyte-derived proteins are involved in the maintenance of energy homeostasis, insulin action, glucose and lipid metabolism and therefore sytemic levels were analyzed before and 2 h after glucose uptake. Because most studies focus on overweight probands or patients that may not reflect the normal situation, plasma levels of several adipose tissue secreted proteins were analyzed in the fasted state and 2 h after the ingestion of 75 g glucose in young, slim and highly insulin-sensitive adults.

In contrast to some studies that describe an induction or no alterations of leptin [2-4], systemic leptin was found reduced 2 h after glucose ingestion to 85 ± 8% in our study group and this is in accordance with the findings of Masuo et al. [5], where insulin-sensitive probands also showed lower leptin levels. Leptin did not change in insulin-resistant subjects in their study [5] indicating an altered response of systemic leptin in insulin-resistant probands. Leptin is suggested to increase insulin sensitivity or to cause insulin resistance [17] and obesity is associated with leptin resistance and a decreased transport of leptin across the blood-brain barrier [18]. Leptin appears to exert its function mainly in the hypothalamus. Leptin increases serotonin turnover by inhibition of nitric oxide (NO) synthesis in the brain and endogenous hypothalamic serotonin (5-hydroxytryptamine) is well described to control appetite and caloric intake [19]. Injection of leptin into the ventromedial hypothalamus increases glucose uptake and decreases hepatic glycogen without any alterations of plasma insulin or glucose levels suggesting that peripheral effects of leptin on glucose metabolism are mediated by the central nervous system [20]. Bradykinin and NO were identified to be involved in leptin-mediated glucose uptake in skeletal muscle [21].

Leptin is not suggested to regulate short-term food intake and central administration of leptin in monkeys reduced food intake the following day [22]. Therefore reduced circulating leptin after carbohydrate ingestion may be an important factor in controlling body weight in slim adults.

In contrast to leptin, adiponectin and resistin were not altered 2 h after glucose uptake and this is in agreement with recent studies [10,11] indicating that systemic adiponectin and resistin are not influenced upon acute hyperglycemia and/or hyperinsulinemia. Adiponectin enhances insulin signaling by reducing p70 S6 kinase-mediated serine phosphorylation of IRS-1 [23] but systemic adiponectin levels are not influenced by carbohydrate uptake. The mRNA expression of the adiponectin receptors AdipoR1 and AdipoR2 is reduced by refeeding in mice and by insulin in-vitro indicating that adiponectin effects are regulated at the receptor level in the postprandial phase [24]. Resistin is regarded as a proinflammatory protein and was recently shown to block insulin signaling in human hepatocytes [25] but al least systemic resistin is not influenced by glucose uptake in insulin-sensitive probands.

The adiponectin paralog CORS-26 was shown to be highly abundant in human plasma for the first time whereas it was not detected in mouse serum [26]. Furthermore, similar to adiponectin, CORS-26 circulates as a HMW complex that is converted to a lower molecular weight form by denaturating conditions. Systemic CORS-26 was similar before and after glucose uptake although immunoblot analysis may only be suitable to detect changes of at least 20% and a suitable ELISA is needed to further investigate systemic CORS-26. So far the physiological role of CORS-26 is unclear. *E. coli *produced CORS-26 stimulates the proliferation of chondrogenic precursors and chondrocytes thereby regulating chondrogenesis and cartilage development [27]. In contrast to adiponectin that is only synthesized by adipocytes, CORS-26 is also expressed in kidney cells and monocytes [12]. Nevertheless CORS-26 may exert similar functions as adiponectin, and antiinflammatory effects [12] and the formation of HMW complexes, by both proteins further supports this hypothesis. Therefore the effect of recombinant CORS-26 on insulin signaling has to be investigated to further evaluate the physiological role of this protein.

Omentin, initially identified as intelectin-1 expressed in intestinal paneth cells [28,29], is highly abundant in the stroma vascular fraction of omental adipose tissue [16]. Omentin was detected in the sera of three donors and the concentration was determined to be 100 ng to 1 μg/ml [16]. This protein recognizes galactofuranose and plays a role in the recognition of bacteria-specific components in the host [30]. In the current study omentin was easily detected in plasma by immunoblot but levels were not markedly influenced upon glucose uptake nor related to the gender of the donor. Omentin enhances insulin-stimulated glucose uptake in subcutaneous and visceral human adipocytes in-vitro [16] and therefore systemic omentin may influence postprandial glucose levels. However, systemic omentin levels did not correlate to postprandial blood glucose indicating that at least circulating omentin is not directly associated with postprandial glucose levels in vivo.

## Conclusion

Postprandial hyperglycemia is associated with lower circulating leptin whereas resistin and adiponectin are not altered in slim, insulin-sensitive probands. CORS-26 and omentin are highly abundant in human plasma and are not severely affected by glucose uptake, although this has to be confirmed with a more quantitative method. Future studies will have to further elucidate the biological relevance of these adipocytokines in glucose homeostasis.

## Abbreviations

Body mass index (BMI), Collagenous repeat-containing sequence of 26-kDa protein (CORS-26), high-molecular weight (HMW), Homeostasis Model Assessment (HOMA), low-molecular weight (LMW), oral glucose tolerance test (OGTT).

## Declaration of competing interests

The author(s) declare that they have no competing interests.

## Authors' contributions

SW performed the OGTTs and carried out immunoassays and immunoblots, MN and JW carried out immunoassays, AS participated in the design of the study, CB conceived of the study, and participated in its design and coordination.

## References

[B1] Koerner A, Kratzsch J, Kiess W (2005). Adipocytokines: leptin--the classical, resistin--the controversical, adiponectin--the promising, and more to come. Best Pract Res Clin Endocrinol Metab.

[B2] Bougoulia M, Tzotzas T, Efthymiou H, Koliakos G, Konstantinidis T, Triantos A, Krassas GE (1999). Leptin concentrations during oral glucose tolerance test (OGTT) in obese and normal weight women. Int J Obes Relat Metab Disord.

[B3] Corica F, Corsonello A, Ientile R, De Gregorio T, Malara A, Artemisia A, Buemi M (2001). Leptin and norepinephrine plasma concentrations during glucose loading in normotensive and hypertensive obese women. Am J Hypertens.

[B4] Cakir M, Sari R, Tosun O, Karayalcin U (2005). Leptin response to oral glucose tolerance test in obese and nonobese premenopausal women. Endocr Res.

[B5] Masuo K, Katsuya T, Ogihara T, Tuck ML (2005). Acute hyperinsulinemia reduces plasma leptin levels in insulin-sensitive Japanese men. Am J Hypertens.

[B6] Okamoto Y, Kihara S, Funahashi T, Matsuzawa Y, Libby P (2006). Adiponectin: a key adipocytokine in metabolic syndrome. Clin Sci (Lond).

[B7] Neumeier M, Weigert J, Schaffler A, Wehrwein G, Muller-Ladner U, Scholmerich J, Wrede C, Buechler C (2006). Different effects of adiponectin isoforms in human monocytic cells. J Leukoc Biol.

[B8] Tsao TS, Murrey HE, Hug C, Lee DH, Lodish HF (2002). Oligomerization state-dependent activation of NF-kappa B signaling pathway by adipocyte complement-related protein of 30 kDa (Acrp30). J Biol Chem.

[B9] Pajvani UB, Hawkins M, Combs TP, Rajala MW, Doebber T, Berger JP, Wagner JA, Wu M, Knopps A, Xiang AH, Utzschneider KM, Kahn SE, Olefsky JM, Buchanan TA, Scherer PE (2004). Complex distribution, not absolute amount of adiponectin, correlates with thiazolidinedione-mediated improvement in insulin sensitivity. J Biol Chem.

[B10] Lewandowski KC, Szosland K, O'Callaghan C, Tan BK, Randeva HS, Lewinski A (2005). Adiponectin and resistin serum levels in women with polycystic ovary syndrome during oral glucose tolerance test: a significant reciprocal correlation between adiponectin and resistin independent of insulin resistance indices. Mol Genet Metab.

[B11] Osei K, Gaillard T, Schuster D (2005). Plasma adiponectin levels in high risk African-Americans with normal glucose tolerance, impaired glucose tolerance, and type 2 diabetes. Obes Res.

[B12] Weigert J, Neumeier M, Schaffler A, Fleck M, Scholmerich J, Schutz C, Buechler C (2005). The adiponectin paralog CORS-26 has anti-inflammatory properties and is produced by human monocytic cells. FEBS Lett.

[B13] Kusminski CM, McTernan PG, Kumar S (2005). Role of resistin in obesity, insulin resistance and Type II diabetes. Clin Sci (Lond).

[B14] Banerjee RR, Rangwala SM, Shapiro JS, Rich AS, Rhoades B, Qi Y, Wang J, Rajala MW, Pocai A, Scherer PE, Steppan CM, Ahima RS, Obici S, Rossetti L, Lazar MA (2004). Regulation of fasted blood glucose by resistin. Science.

[B15] Schaffler A, Buchler C, Muller-Ladner U, Herfarth H, Ehling A, Paul G, Scholmerich J, Zietz B (2004). Identification of variables influencing resistin serum levels in patients with type 1 and type 2 diabetes mellitus. Horm Metab Res.

[B16] Yang RZ, Lee MJ, Hu H, Pray J, Wu HB, Hansen BC, Shuldiner AR, Fried SK, McLenithan JC, Gong DW (2006). Identification of omentin as a novel depot-specific adipokine in human adipose tissue: possible role in modulating insulin action. Am J Physiol Endocrinol Metab.

[B17] Harris RB (2000). Leptin--much more than a satiety signal. Annu Rev Nutr.

[B18] Burguera B, Couce ME, Curran GL, Jensen MD, Lloyd RV, Cleary MP, Poduslo JF (2000). Obesity is associated with a decreased leptin transport across the blood-brain barrier in rats. Diabetes.

[B19] Calapai G, Corica F, Corsonello A, Sautebin L, Di Rosa M, Campo GM, Buemi M, Mauro VN, Caputi AP (1999). Leptin increases serotonin turnover by inhibition of brain nitric oxide synthesis. J Clin Invest.

[B20] Kamohara S, Burcelin R, Halaas JL, Friedman JM, Charron MJ (1997). Acute stimulation of glucose metabolism in mice by leptin treatment. Nature.

[B21] Shiuchi T, Nakagami H, Iwai M, Takeda Y, Cui T, Chen R, Minokoshi Y, Horiuchi M (2001). Involvement of bradykinin and nitric oxide in leptin-mediated glucose uptake in skeletal muscle. Endocrinology.

[B22] Tang-Christensen M, Havel PJ, Jacobs RR, Larsen PJ, Cameron JL (1999). Central administration of leptin inhibits food intake and activates the sympathetic nervous system in rhesus macaques. J Clin Endocrinol Metab.

[B23] Wang C, Mao X, Wang L, Liu M, Wetzel MD, Guan KL, Dong LQ, Liu F (2007). Adiponectin sensitizes insulin signaling by reducing p70 S6 kinase-mediated serine phosphorylation of IRS-1. J Biol Chem.

[B24] Tsuchida A, Yamauchi T, Ito Y, Hada Y, Maki T, Takekawa S, Kamon J, Kobayashi M, Suzuki R, Hara K, Kubota N, Terauchi Y, Froguel P, Nakae J, Kasuga M, Accili D, Tobe K, Ueki K, Nagai R, Kadowaki T (2004). Insulin/Foxo1 pathway regulates expression levels of adiponectin receptors and adiponectin sensitivity. J Biol Chem.

[B25] Zhou L, Li Y, Xia T, Feng S, Chen X, Yang Z (2006). Resistin overexpression impaired glucose tolerance in hepatocytes. Eur Cytokine Netw.

[B26] Akiyama H, Furukawa S, Wakisaka S, Maeda T (2006). Cartducin stimulates mesenchymal chondroprogenitor cell proliferation through both extracellular signal-regulated kinase and phosphatidylinositol 3-kinase/Akt pathways. Febs J.

[B27] Maeda T, Jikko A, Abe M, Yokohama-Tamaki T, Akiyama H, Furukawa S, Takigawa M, Wakisaka S (2006). Cartducin, a paralog of Acrp30/adiponectin, is induced during chondrogenic differentiation and promotes proliferation of chondrogenic precursors and chondrocytes. J Cell Physiol.

[B28] Schaffler A, Neumeier M, Herfarth H, Furst A, Scholmerich J, Buchler C (2005). Genomic structure of human omentin, a new adipocytokine expressed in omental adipose tissue. Biochim Biophys Acta.

[B29] Komiya T, Tanigawa Y, Hirohashi S (1998). Cloning of the novel gene intelectin, which is expressed in intestinal paneth cells in mice. Biochem Biophys Res Commun.

[B30] Tsuji S, Uehori J, Matsumoto M, Suzuki Y, Matsuhisa A, Toyoshima K, Seya T (2001). Human intelectin is a novel soluble lectin that recognizes galactofuranose in carbohydrate chains of bacterial cell wall. J Biol Chem.

